# Long-term psychological impact of the pandemic COVID-19: Identification of high-risk groups and assessment of precautionary measures five months after the first wave of restrictions was lifted

**DOI:** 10.1371/journal.pgph.0002847

**Published:** 2024-02-23

**Authors:** Ioulia Solomou, Flora Nikolaou, Michalis P. Michaelides, Fofi Constantinidou

**Affiliations:** 1 Department of Psychology, University of Cyprus, Nicosia, Cyprus; 2 Center for Applied Neuroscience, University of Cyprus, Nicosia, Cyprus; PLOS: Public Library of Science, UNITED STATES

## Abstract

Critical facets of our lives have been disrupted by the COVID-19 outbreak for almost three years. During this time, there has been a lot of clinical and research interest in issues related to mental health. However, few have examined the pandemic’s long-term psychological effects. The aims of the present study were to assess the long-term psychological impact of the pandemic COVID -19, five months after the first wave restriction measures were lifted, to identify high-risk groups and to address the use of precautionary measures (PM). Information about sociodemographic characteristics, mental health, coping mechanisms, and compliance with precautionary measures (PM) were all gathered in Cyprus through an anonymous online survey. The poll was completed by 1128 people (73% of whom were female). For the purposes of the present study, descriptive statistics and structural equation modeling were used. 32.3% of participants experienced moderate-to-severe anxiety symptoms, where 16.4% and 23% reported moderate-to-severe depression and post-traumatic stress symptoms respectively. Lower levels of anxiety, depression, and post-traumatic stress symptoms as well as greater compliance to PM were linked to higher levels of resilience. Additionally, subgroups of participants, at a higher risk for negative psychological effects were identified, such as women and young adults. Our findings demonstrate the long-lasting effects of the COVID-19 pandemic on mental health and provide guidance on how to deal with similar situations. It also raises questions about the concurrent effects on people with the long COVID syndrome.

## Introduction

In early 2020 people around the world were confronted with the now known Coronavirus 2019 (COVID -19). The CΟVID -19, was classified as life-threatening from the outset as it could cause severe health consequences such as respiratory arrest, acute respiratory distress syndrome, and cardiac arrhythmias in certain at-risk groups [1, 2]. Considering the high contagion rate, the absence of effective medications or vaccines, the high rates of hospitalizations and deaths, and the overwhelming demands that strained the national health care systems across the globe, most countries implemented restriction measures to curtail the spread the disease. Cyprus, implemented very strict restrictiοns in movement, including a six week lockdown starting in mid-March 2020 through the beginning of May 2020. This new reality was reportedly a major stressor as it disrupted important aspects of daily life, created financial uncertainty and affected quality of life [3–6].

The unprecedented disruptive forces of the pandemic, gave rise to a plethora of online surveys set to examine individual risk factors leading to identification of vulnerable groups likely to suffer significant psychological effect during the lockdown (e.g. anxiety and depression symptoms). Health care workers [7, 8], younger adults [4, 9] college students [4, 9–12], and unemployed individuals [4, 13] were at a greater risk for developing psychological distress.

Gender differences have also been reported, with women at higher risk than men of developing negative psychological symptoms due to the pandemic [4, 14–16]. Related research have reported that women showed elevated levels of feelings of loneliness than men during the quarantine period [17].

Another important and expected high-risk group facing more psychological symptoms are those with a history of psychological distress [4, 18]. Not only are these individuals more psychologically vulnerable, but they are also prone to have smaller sοcial networks groups, and the restriction measures due to the pandemic may have added additional stressors for them [19].

Very few studies have investigated resilience and coping mechanisms during the initial phase of the pandemic when severe measures were applied and the period immediately following. What we do know about that period (which can certainly make an important contribution to the evaluation of today’s mechanisms and whether time has influenced the way the mechanisms are used), is that maladaptive coping behaviors (e.g., avoidance thinking, substance and alcohol abuse, overeating) were assοciated with increased mental problems and lower quality of life (QOL), while adaptive coping mechanisms (e.g., positive thinking, humor, sports training) were assοciated with lower depression symptomatology and better QOL [20–24].

Regarding coping mechanisms, differences between groups, e.g., by gender, were also found. Women, for example, exhibited more maladaptive behaviors to cope with pandemic anxiety compared with men, such as excessive time spent on online applications and overeating [22, 24, 25]. Age also appears to be one of the most fundamental demographic elements related to coping mechanisms; young people did not appear to cope well with the pandemic compared to older adults [5, 24]. Younger people were more prone to behaviors such as alcohol consumption, disregard for government regulations, lack of social distancing, and general noncompliance with precautions against COVID -19 [26, 27]. Meladaptive coping mechanisms, such as the use of alcohol, could cause a vicious cycle of dependence and their continued use reinforces the problems [22, 25]. Therefore, it is important today that research be based on recognizing patterns that prevailed during the period of strict measures, as well as immediately thereafter, to be able to identify patterns that may have changed or remained the same.

Compliance with precautionary measures against COVID-19 also appears to be linked with mental health effects. Specifically, the implementation of the precautionary measures has protective psychological effects, especially against depression symptomatology [4, 16]. However, very high adherence to the personal hygiene safety measures such as hand washing, use of disinfectant, wiping down knobs and surfaces, has been associated with higher levels of anxiety [4].

Currently, three years after the onset of the pandemic, the number of individuals who sustained COVID-19 exceeds 760 million, in contrast to around 850 thousands during the first wave of the pandemic [28]. Several virus mutations and multiple efforts to curtain the additional several waves of the pandemic have taken place globally. Yet, the world is still facing the societal, financial and health consequences. In addition, a large percentage of patients who recovered from COVID-19 (ranging from 10–20% depending on the study & methodology implemented), are facing long term health effects of the disease (i.e. long-COVID or post-acute COVID syndrome), including psychological and emotional problems [29]. Therefore, characterizing the psychological effects of COVID-19 during the initial waves of the pandemic could help develop targeted public health programs and prevent long-lasting mental health effects of COVID-19. explain some of the.

In summary, a variety of demographic variables could increase the risk for psychological difficulties and also influence individual coping mechanisms. While multiple studies have reported the immediate psychological effect of the COVID-19 outbreak and lockdown, there is a need for continuous evaluation of the long term effects of the pandemic on mental health. Therefore, the present study contributes to the growing body of evidence on the long-term psychological impact of the COVID-19 pandemic in the general population. Specifically, this study explored the associations between the psychosocial impact of COVID-19 pandemic, the associated anxiety and depression symptoms, post-traumatic stress symptoms, coping mechanisms, and compliance with precautionary measures through a structural equation model. It was hypothesized that the elevated depression and anxiety symptoms documented during the first COVID-19 wave in the general public would have subsided several months afterwards and the epidemiological picture would have improved. Based on prior research, we also hypothesize that women and young adults will exhibit a greater vulnerability to experience psychological effects over the long term due to the impact of COVID-19.The findings contribute to the literature by characterizing the long lasting effects of the pandemic on mental health and provide important information to current efforts aiming to identify risk factors for persistent psychological effects associated with the post-covid condition.

## Materials and methods

The present cross-sectional study adhered to the same methodology of the Solomou and Constantinidou (2020).

### Participants and procedure

On the online survey platform Research Electronic Data Capture (REDCap), participants filled out a self-report questionnaire [30]. The poll was brief (up to 10 minutes) and bilingual to increase participation (Greek and English). Informed consent was required before the survey was conducted. Five months after the lifting of lockdown measures and movement restrictions, data were gathered over a one-month period from October 18 to November 18, 2020. The Cyprus Bioethics Committee granted ethical approval with the following number: EΕΒΚ ΕΠ 2020.01.54

The final sample included 1128 participants (72.9% of whom were female), with the bulk of participants being between the ages of 18 and 28 (43.3%), followed by the ages of 30–39 (20.1%), 40–49 (19.6%), 50–59 (11.6%), and 60 and over (5.4%). The final sample included in the analysis included only complete data. Table 1 displays the sociodemographic details of the final sample.

**Table 1:** Participants’ demographic information.

Variable	n (%)
**Sex**	
Female	822(72.9)
Male	306(27.1)
**Age**	
18–29	488(43.3)
30–39	227(20.1)
40–49	221(19.6)
50–59	131(11.6)
60+	61(5.4)
**Living Status**	
Alone	224(21.6)
With others	884(78.4)
**Level of Education**	
Primary Education	7(.6)
Secondary Education	319(28.3)
Tertiary Education	756(67)
None	46(4.1)
**Student Status**	
Yes	590(52.3)
No	538(47.7)
**Employed status**	
Yes	687(60.9)
No	441(39.1)
**Time spent on social media about COVID-19?**	
Up to 10 minutes	706(62.6)
30–40 minutes	235(20.8)
At least one hour or more	71(6.3)
Not at all	116(10.3)
**Do you believe that the coronovirus is made into a big deal and is given too much attention with no particular reason?**	
Yes	405(35.9)
No	723(64.1)
**Are you satisfied with how the government handled the pandemic in the country you are currently residing?**	
Yes	408(36.2)
No	337(29.9)
Neither satisfied or dissatisfied	383(34)
**Psychiatric History**	
Yes	185(16.4)
No	943(83.6)
**Do you belong in a COVID-19 vulnerable group?**	
Yes	162(14.4)
No	966(85.6)

### Survey and measures

The questionnaire consisted of six sections: (1) Sοciοdemographic and medical history, (2) COVID -19 related concerns, (4) mental health measures, (5) assessment of coping mechanisms, and (6) adherence to precautions.

### Sοciοdemographic and medical history

Sociodemographic information on gender, age, education level, housing situation, employment status, and student status was collected in the first section. In addition, questions were asked about medical history, which included previous anxiety or depression diagnoses and membership in a CΟVID -19 risk group.

### COVID -19 related concerns

Other questions related to COVID -19 included whether they were satisfied with the government’s protective measures, how much time they spent on social media with COVID -19, and whether they think coronavirus is a big deal.

### Mental health measures

Anxiety levels as measured by the Generalised Anxiety Disorder 7 scale (GAD-7) asks respondents to rate their own anxiety on a 4-point Likert scale [31]. The seven main symptoms of generalised anxiety disorder questioned how often participants were disturbed by them in the last two weeks. The total score could be classified as normal, mild, moderate, or severe anxiety [32]. Reliability resulted in a Cronbach’s alpha at 0.93.

Depression measured with Patient Health Questionnaire 9 (PHQ-9). The frequency with which the nine primary symptoms of depression disturbed subjects throughout the previous two weeks was questioned [33]. Normal, mild, moderate and major depression symptomatology clusters composed of the total score [34]. Reliability resulted in a Cronbach’s alpha at 0.90.

Posttraumatic stress Measured with Impact of Event Scale with Modifications for CΟVID -19 (IES-CΟVID19). This is a self-report questionnaire consisting of 15 items developed to evaluate posttraumatic stress symptoms related to the impact of the COVID -19 outbreak [35]. Seven items measure intrusion and eight items measure avoidance behaviors. The scale was translated frοm English tο Greek by our team using forward and backward translation. Individuals were asked how often they had been bothered in the past 7 days by each of the 15 statements related to the situation at COVID -19. Higher score reflects higher stress about COVID -19. The total score can be interpreted into 0–8 subclinical posttraumatic symptoms, 9–25 mild posttraumatic symptoms, 26–43 moderate, 44–75 severe posttraumatic symptoms. The Cronbach’s α was 0.91.

### Assessment of coping mechanisms

To assess coping mechanisms, the Brief Resilient Coping Scale (BRCS) was used. It consists of 4-items assessing the tendency to cope with stress in a highly adaptive manner on a 5-point Likert scale [36]. The Cronbach’s α was 0.74.

### Adherence to precautions

To assess adherence to precautions (PM), we used the 13-question scale developed by Solomou and Constantinidou (2020). Subjects responded how often they adhered to precaution measures in the past 2 weeks on a 5-point likert scale. Questions 4, 5, 6, 7, 8, 9, and 10 address "outdoor precautions," while questions 1, 2, 3, 11, 12, and 13 address "personal hygiene and indoor precautions" [4]. The Cronbach’s α was 0.87.

### Analysis

Initially, SPSS Statistic 25.0 was used to examine descriptive statistics (means, standard deviations, and correlation analysis between outcome measures); SPSS AMOS 20.0 was used for latent variable analysis.

A separate confirmatory factor analysis (CFA) was performed for each outcome measure to validate the measurement model and see whether the developers’ suggested factor structure was a satisfactory fit with the existing data. Using χ^2^ test and other evaluation indices, such as the comparative fit index (CFI), root mean square error of approximation (RMSEA), Tucker-Lewis index (TLI), and standardised mean residual, the goodness of fit of the CFA models was evaluated (SRMS). The following cutoffs were used to determine what constitutes an acceptable level of model fit: CFI .90, RMSEA .08, TLI .90, and SRMR .08. In order to determine latent relationships between outcomes, the top CFA model for each outcome measure was simultaneously entered into a correlated latent component model. In order to investigate the effects of sociodemographic variables on important outcome indicators, a structural equation model (SEM) was created.

## Results

### Descriptive statistics on outcome measures

Table 2 displays the scale means and standard deviations. According to the GAD-7 score distribution, 412 participants (36.5%) scored in the healthy range (0–4), 352 (31.2%) scored in the mild anxiety range (5–9), 202 (17.9%) scored in the moderate anxiety range (10–14), and 162 (14.4%) scored in the severe anxiety range (15–21). In the PHQ, 404 (35.6%) people had a score that was within a healthy range (score: 0–4). A large prοpοrtion of the sample, 539 (47.8%), repοrted mild depressive symptomatοlοgy (scοre: 5–14), while 109 (9.7%) respοndents repοrted moderate depressive symptomatology (scοre: 15–19) and 76 (6.7%) respοndents reported severe depressive symptomatοlogy (score: 20–27). On the IES-COVID19, a large number of respondents, 449 (39.8%), were in the healthy range (score: 0–8). 419 (37.1%) repοrted mild IES-COVD19 symptοms (score: 9–25), while 218 (19.3%) respοndents repοrted moderate-severe IES-COVID19 symptoms (score: 26–43) and 42 (3.7%) reported severe symptοms (scοre: 44–75). On the BRCS, 347 participants (30.8%) were classified as having low resilience (score: 4–13); a large proportion of participants, 534 (47.3%), reported moderate resilience and coping mechanisms, while the remainder of participants, 247 (21.9%), reported high resilience.

**Table 2:** Correlations between the five objectives variables.

Measure	Mean	St. Dev.	1	2	3	4
**1. Precautionary Measures**	45.75	9.14				
**2. BRCS**	14.57	2.84	.128**			
**3. IES-COVID 19**	15.87	14.91	.084**	-.122**		
**4. GAD-7**	7.55	5.68	.042	-.307**	.607**	
**5. PHQ**	7.97	6.18	-.043	-.295**	.578**	.782**

*p < .05

**p < .01

*** p < .001

Pearson’s r correlations between outcome measures are shown in Table 2. There were strong positive assοciations between anxiety, depression symptomatology, and IES-COVID 19 symptom reports. The three mental health outcomes were weakly and negatively correlated with resilience. Preventive measures scores were very weakly and positively cοrrelated with resilience and IES-COVID 19.

### Confirmatory factor analysis

The one-factor structure model of the GAD-7 provided a poor fit according to the goodness of fit indices, χ^2^(14) = 135.375, p < .001; CFI  =  0.98; RMSEA  =  0.088; TLI = 0.97 and SRMR  =  0.022. Examination of the modification indices recommended a covariance between residuals of Items 1 and 5. The revised model gave a better model fit, χ^2^ (13) = 78.035, p < .001; CFI = 0.99; RMSEA = 0.06; (90% CI: 0.053 to 0.081); TLI = 0.98 and SRMR = 0.0175.

The one-factor model of the PHQ-9 had reasonable fit indices except for the RMSEA χ^2^ (27) = 295.319, p < .001; CFI  =  0.94; RMSEA  =  0.094; (90% CI: 0.084 to 0.104); TLI = 0.93 and SRMR  =  0.039. Examination of the modification indices suggested residual covariances between Items 1 and 2, Items 6 and 9 and between Items 7 and 8. The correlated residuals were expected since those items measure cognitive symptoms. The revised model gave a better model fit, χ^2^ (24) = 160.168, p < .001; CFI = 0.97; RMSEA = 0.07; (90% CI: 0.061 to 0.082); TLI = 0.96 and SRMR = 0.0175.

For the IES-COVID19, we compared the fit indices of a single-factor model, a two-factor model, and a bifactor model with two specific factors for intrusion and avoidance. The fit indices of the one factor, χ^2^ (90) = 1461.457, p < .001; CFI  =  0.84; RMSEA  =  0.116; (90% CI: 0.111 to 0.122); TLI = 0.81 and SRMR  =  0.0747, and two-factor model, χ^2^ (89) = 1287.767, p < .001; CFI  =  0.86; RMSEA  =  0.109; (90% CI: 0.104 to 0.115); TLI = 0.83 and SRMR  =  0.039, were poorer than those of the bifactor model, χ^2^ (74) = 489.419, p < .001; CFI  =  0.95; RMSEA  =  0.07; (90% CI: 0.065 to 0.077); TLI = 0.93 and SRMR  =  0.035.

The one-factor structure model of the BRCS was not good according to the goodness of fit indices, χ^2^ (2) = 43.046, p < .001; CFI  =  0.95; RMSEA  =  0.135 (90% CI: 0.102 to 0.171); TLI = 0.87 and SRMR  =  0.038. Examination of the modification indices recommended correlation between residuals of Items 1 and 4; both items talk about behavioral type coping mechanisms. The revised model gave a better model fit, χ^2^ (1) = 4.113, p = 0.04; CFI = 0.99; RMSEA = 0.05 (90% CI: 0.008 to 0.109); TLI = 0.98 and SRMR = 0.099.

The one-factor structure model of the PM was not acceptable according to the goodness of fit indices, χ^2^ (90) = 925.552, p < .001; CFI  =  0.82; RMSEA  =  0.108; (90% CI: 0.102 to 0.115); TLI = 0.78 and SRMR  =  0.068. Examination of the modification indices recommended covariances between residuals of Items 1 and 2 as well as Items 8 and 9. Then, we compared the fit indices of the one-factor model, two-factors model, bifactor model with two specific factors: “Personal hygiene and Indoors-related precautionary measures” and “Outdoors-related precautionary measures”. The fit indices of the one factor, χ^2^ (63) = 626.070, p < .001; CFI  =  0.88; RMSEA  =  0.089; (90% CI: 0.08 to 0.09); TLI = 0.85 and SRMR  =  0.058, and the two-factor model, χ^2^ (63) = 967.281, p < .001; CFI  =  0.801; RMSEA  =  0.113; (90% CI: 0.107 to 0.119); TLI = 0.761 and SRMR  =  0.205 were poorer than those of bi-factor model, χ^2^ (50) = 267.213, p < .001; CFI  =  0.95; RMSEA  =  0.062; (90% CI: 0.054 to 0.068); TLI = 0.928 and SRMR  =  0.033. None of the observed variables loaded significantly on the “Personal hygiene and Indoors-related precautionary measures” specific factor, so we proceeded with a bifactor model with one specific factor. This model had an adequate fit, χ^2^ (56) = 309.882, p < .001; CFI  =  0.94; RMSEA  =  0.063; (90% CI: 0.057 to 0.070); TLI = 0.925 and SRMR  =  0.036, therefore, we selected the latter model for subsequent analyses. Fit indices for all revised models appear on Table 3.

**Table 3:** Best fitting measurement models of outcome variables.

Model	χ^2^	df	CFI	RMSEA	90%CI RMSEA		TLI	SRMR
					*LL*	*UL*		
**GAD-7: Single factor with 1 error covariance**	78.04***	13	0.99	0.068	0.05	0.08	0.98	0.01
**BRCS: Single factor with 1 error covariance**	4.12	1	0.99	0.05	0.01	0.11	0.98	0.01
**PHQ: Single factor with 3 error covariances**	160.17***	24	0.97	0.07	0.06	0.08	0.96	0.03
**IES-COVID: Bifactor with 2 specific factors**	489.42***	74	0.95	0.07	0.06	0.08	0.93	0.04
**Precautionary Measures: Bifactor with one specific factor and 2 error covariances**	309.88***	56	0.95	0.06	0.06	0.07	0.93	0.04
**Final Measurement Model**	2979.96***	1041	0.93	0.04	0.039	0.042	0.92	0.044

*p < .05

**p < .01

*** p < .001

### Latent associations

After ensuring that the psychometric properties of each outcome measure were satisfactory, we moved to a measurement model with latent associations among the five scales. The overall fit of the model was adequate, χ^2^ (1041) = 2979.959, p < .001; CFI = 0.93; RMSEA = 0.04; (90% CI: 0.039 to 0.042); TLI = 0.92; and SRMR = 0.044]. Consistent with the observed correlations, the latent construct of anxiety, measured by GAD, was significantly associated with higher scores on PHQ (r = .86, p≤ .01), IES (r = .65, p≤ .01), and lower scores on coping mechanisms, measured by BRCS (r = -.44, p≤ .01). The latent construct IES-COVID19 was also significantly associated with higher scores on PHQ (r = .64, p≤ .01) and significantly associated with lower scores on coping mechanisms measured by BRCS (r = -.26, p≤ .01). The latent construct BRCS was also significantly associated with higher scores on PM (r = .15, p≤ .01) and lower scores on PHQ (r = -.42, p≤ .01).

### Structural equation model

**Fig 1:**
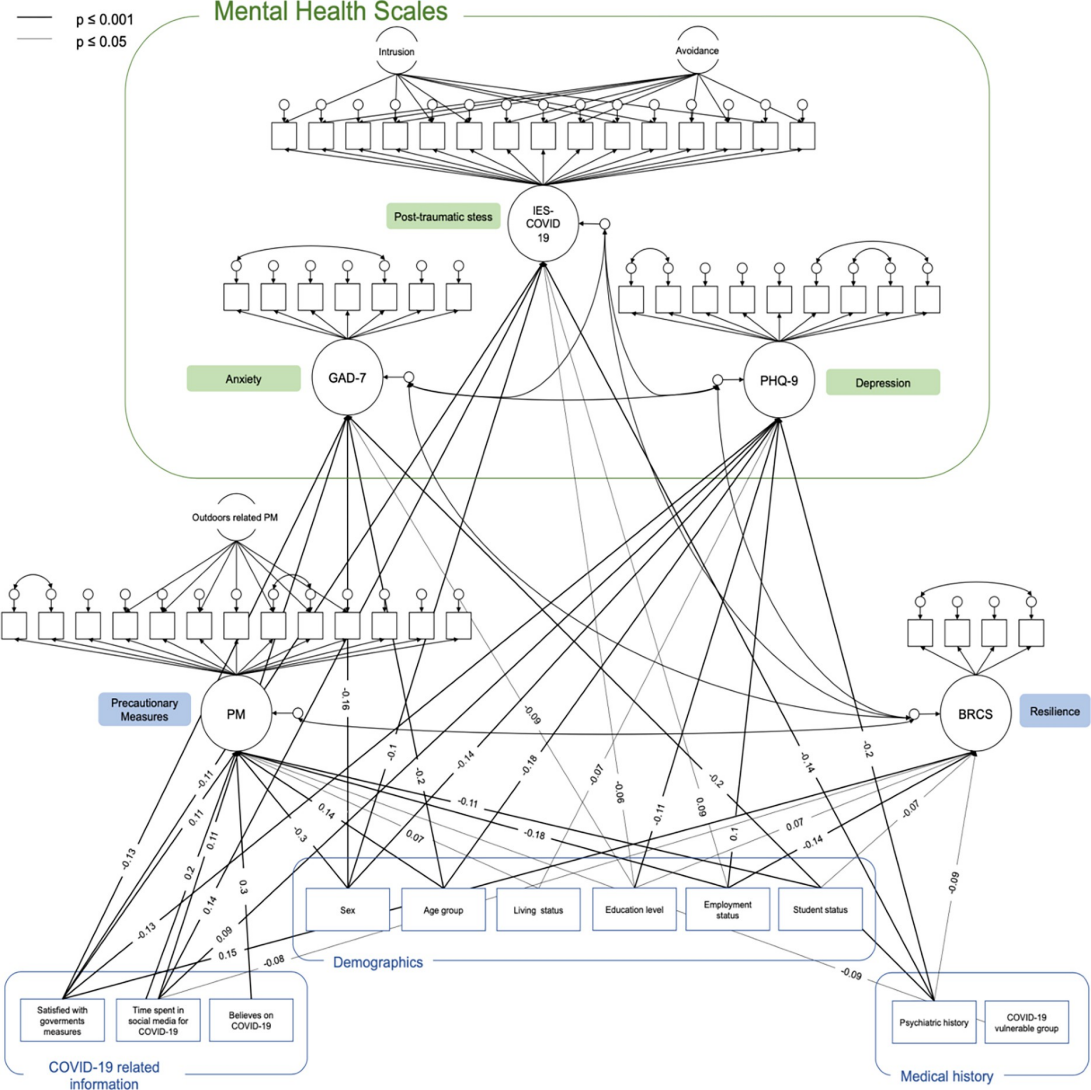
SEM with statistically significant associations between sociodemographic information, COVID-19 related information and the outcome latent factors.

The SEM examining the relationships between the sociodemographic information and the latent outcome factors, is shown in Fig 1. The model showed acceptable fit, χ^2^ (1572) = 5426.616, p < .001; CFI = 0.90; RMSEA = 0.047; (90% CI: 0.045 to 0.048); TLI = 0.9; and SRMR = 0.05.

Fig 1 shows the statistically significant regression coefficients between the sociodemographic information and the latent outcome factors. Among demographic characteristics, gender was directly associated with GAD -7 (β = -.16, p≤ .01), PHQ-9 (β = -.14, p≤ .01), IES-COVID19 (β = -.10, p≤ .01), and PM (β = -.30, p≤ .01) scores. Women reported higher mental health scores and were more likely to adhere to precautions than men. Younger age was associated with lower adherence to PM (β = .14, p≤ .01) and higher GAD -7 (β = -.20, p≤ .01) and PHQ-9 (β = -.18, p≤ .01) scores. In addition, housing status was positively related to PM (β = .07, p≤ .05) and negatively related to depression (β = -.07, p≤ .05); those who lived alone felt more depressed and adhered less to cautions than those who lived with others. Education level had a significant negative effect on GAD -7 (β = -.09, p≤ .05), PHQ-9 (β = -.11, p≤ .001), IES-COVID19 (β = -.06, p≤ .05), and a positive effect on BRCS (β = .07, p≤ .05). Those who reported more education were less psychologically impaired and more resilient than those who thought they were less educated. Unemployment was associated with higher PHQ-9 (β = .10, p≤ .001) and IES-COVID19 (β = .09, p≤ .05) scores, lower compliance with PM (β = -.18, p≤ .001), and lower BRCS (β = -.14, p≤ .001) scores. Student status was directly related to higher BRCS scores (β = -.07, p≤ .05) and higher compliance with PM (β = -.11, p≤ .001).

In terms of COVID -19 related information, higher satisfaction with the way the government is handling the pandemic was associated with higher compliance with PM (β = .11, p≤ .001) and higher BRCS (β = .15, p≤ .001) scores and associated with lower GAD -7 (β = -.13, p≤ .001), PHQ-9 (β = -.13, p≤ .001), and IES-COVID19 (β = -.11, p≤ .001) scores. In addition, those who spent more time daily on social media via COVID -19 reported higher GAD -7 (β = .11, p≤ .001), PHQ-9 (β = .10, p≤ .001), and IES-COVID19 (β = .14, p≤ .001) scores; they were also more compliant with PM (β = .20, p≤ .001). Those who believed coronavirus was a big deal were also more compliant with PM (β = .3, p≤ .001)

Psychiatric history correlated with higher GAD -7 (β = -.20, p≤ .001), PHQ-9 (β = -.20, p≤ .001), and IES-COVID19 (β = -.14, p≤ .001) symptoms and with lower BRCS (β = -.09, p≤ .05) scores. Those belonging to COVID -19 at-risk groups were significantly associated with higher compliance with PM (β = -.09, p≤ .05) only.

## Discussion

In an effort to bring the pandemic under control, governments around the world took unprecedented restrictive measures to limit movement and promote social distancing. In Cyprus, the strict lockdown measures were implemented in mid March 2020 and lasted approximately 8 weeks. Cyprus was praised internationally for the effectiveness of its pandemic response [37]. The results of the current study provide important insights on how people adapted 5 months after the removal of restrictive measures and shed light on long-term mental health problems.

Among the most important findings of the present study were the elevated scores for anxiety and depression. Specifically, 32.3% reported moderate to severe anxiety symptoms and 16.4% of the total sample reported moderate to severe depression symptoms. This trend indicates an impressive increase compared to a previous study with the same research methodology that occurred during lockdown, in which 23.1% and 9.2% of the sample reported moderate to severe anxiety and depression symptomatology, respectively [4]. Posttraumatic stress symptoms were also significantly elevated, as 23% of individuals in the present study reported moderate-severe symptoms of PTSD. The presence of mental morbidity was associated with younger age, women and psychiatric history. Coping mechanisms were also associated with individuals’ mental health status and were influenced by several sociodemographic variables such as employment and student status. Our study found that individuals who adhered to precautionary measures reported better coping with the pandemic compared to those who did not adhere to these measures. Additionally, those who expressed greater concern about the government’s handling of the pandemic were more likely to adhere to these measures. At the same time, women, the elderly, professionals, and participants who lived with others were more likely to adhere to the PM.

### Anxiety, depression, and PTSD symptomatology and vulnerable groups

This cross-sectional study expand our knowledge of the psychological effect of COVID -19 [4, 11, 16] and raise significant concerns about the adjustment and long-term psychological needs of individuals following a pandemic. As mentioned, anxiety and depression symptomatology appeared to be elevated following the lifting of the strict lockdown, which was implemented in the spring of 2020 [4]. We hypothesized that the anxiety and depression symptomatology observed during the first wave would have subsided. After the first wave and the relaxation of restrictive measures, Cypriots enjoyed a COVID -free summer (with no reported infections for several weeks) and with a few restrictive measures mainly related to operating hours and occupancy capacity of restaurants and clubs. During the period in which we collected the data, there was a small increase in cases and discussions about a possible second wave in late fall. According to our results, 23% of our sample had moderate to severe post-traumatic stress symptomatology regarding the lockdown period.

As expected, the distribution of symptoms varied by gender. Women reported higher scores on all mental health measures. Regardless of conditions, epidemiological research indicates that women are the most vulnerable group to developing mental health problems [4, 14]. Therefore, this conclusion is consistent with these studies.

Another vulnerable group in which intense emotional stress was reported was the younger age group. One possible explanation for the differences on the age axis is that young people in Cyprus have not yet experienced a traumatic event in its entirety compared to their elders. In contrast, the elder Cypriot population was confronted with the invasion of Turkey in 1974, in which their lives were directly threatened, and can easily be classified as a traumatic event to endure [4]. Apart from the hypothesis that young people may not have developed appropriate coping mechanisms, younger people and those who were studying at university have had to adapt to many changes in their lifestyle and develop new skills since the outbreak of the pandemic. In Cyprus, most universities continued to operate remotely and online after the lockdown, which in some cases led to changes in lifestyle and mode of study. Even after the strict restrictive measures were lifted, students still faced these changes and were more vulnerable to feelings of stress and frustration [4, 12, 38].

Other variables such as housing situation and education level were associated with reported mental health outcomes. Specifically, those who lived alone reported more depression symptoms than those who lived with others, although this effect was very weak. This finding contradicts the results of the first wave, as those who lived with others reported more depression [4]. Nonetheless, this specific finding is quite optimistic about the pattern, because in conditions where the obligatory factors of social isolation are absent, people living alone are more prone to symptoms of depression and anxiety [39]. Thus, it appears that the specific pattern returned to the expected over a five-month period. Participants with higher education were alsο repοrted to have fewer symptoms. It could be that those with higher education had more resources and a stable job and were therefore less likely to have financial problems. This finding agrees with previous research that has found a strong association between higher levels of education and lower levels of psychologic distress in the absence of the pandemic [40, 41]

### Mental health and COVID-19 related questions

Continuing on the theme of psychological effects, the findings on questions related to COVID-19 are equally intriguing as the previously reported data. Individuals who expressed satisfaction with their government’s handling of the pandemic were also less emotionally distressed. Satisfaction implies a higher sense of security from the disease, while these individuals also have more trust in the authorities and are more willing to follow government instructions to protect themselves from the virus [42]. Women reported greater dissatisfaction with the gοvernment’s handling of the CΟVID -19 crisis than men [43]. The role of gender in decision-making during the pandemic is an important factor to consider, particularly in light of women’s reported frustration with how the pandemic was handled. Understanding the relationship between gender and decision-making may shed light on why women may be experiencing and maintaining increased mental health problems during this time.

In addition, individuals who spent more time daily on social media to learn about the pandemic reported greater distress. This trend is consistent with previous research showing that media exposure during lockdown increases the risk for depressive symptoms and COVID -19 specific anxiety [25, 44, 45]. Social media outlets were crucial throughout the pandemic as they served as the primary informational channels for COVID-19 (e.g., government measurements, infection rates, deaths due to COVID -19, etc.). However, social media can also be dangerous because of the rise in the number of unfiltered and, occasionally, false messages that can harm mental health [44].

### Compliance with CΟVID -19 precautions

In addition to mandatory and stringent measures to restrict movement, individual precautionary measures were (and are) encouraged to be implemented to reduce transmission of the virus. This study examined how well the community has implemented thirteen key PMs five months after the lockdown was lifted. Results showed a positive association between PM implementation and resilience and coping, which is consistent with previous studies that found an association between higher adherence to PM and lower psychological distress and greater coping [16, 46]

Gender and age differences were alsο fοund with respect to PM adherence. Women and οlder individuals were mοre likely to adhere to PM than men and younger adults. Existing literature on precautions suggests that men and young adults are at higher risk of infection because they are less likely to adhere to the COVID -19 precautions [47, 48] Nevertheless, it is encouraging, as also mentioned by Tοng et al. [49], that those who belonged to COVID -19 vulnerable groups were more likely to adhere to PM, as adherence to such measures was strong negatively associated with the severity of the health effects of CΟVID -19.

It would be a major omission not to also mention that individuals who reported spending more time on social media and felt that COVID -19 was an important issue also showed a high level of engagement with precautions. Although social media carries the risk of bias and increases the likelihood of emotional distress, it appears to be beneficial in the present case, as the more informed individuals were better able to protect themselves than the uninformed [47, 50] High levels of engagement were observed among individuals who reported satisfaction with their government’s pandemic response. The implementation of public health recommendations may have been facilitated by the internal communalization of these individuals, as suggested by Tong et al. [49]. Thus, it is also important to note at this point the need for interventions that correctly inform the public about the effectiveness of preventive measures and the risks. The government can use the existing data to develop programs and campaigns that specifically target the groups least likely to adhere to PMs. In order to control infection, personal hygiene was a crucial component of personal safety, especially when lockdown was released. To stop the transmission of disease and infection, hygiene compliance continues to be a primary public health concern. Governments admonish residents to follow these rules so they can resume their normal lives.

Living status was also related to adherence to PM. Those who lived with others were more likely to adhere to precautions than those who lived alone. This finding is supported by the fact that individuals who live with others are more concerned about infecting themselves or/and those around them, and therefore more likely to adhere to preventive behaviors [4, 47].

### The role of coping mechanisms

During the lockdown, little research has examined vulnerability factors (such as stress tolerance and loneliness; [51]) that may contribute to the choice of coping menisms, as well as how coping mechanisms affect mental health [52]. In the current study, we found a negative correlation between coping strategies psychological distress. According to preliminary research, there is a link between maladaptive coping behaviours (such as avoidance, alcohol and drug use, and overeating) and higher levels of distress [20, 22, 23, 53].

### Implications & limitations

The present study is a cross-sectional study using the same methodology as a previous study conducted at the height of the first COVID-19 wave οf the pandemic and during the first strict lοckdοwn in Cyprus [4]. Both studies collected anonymous data to maximize the accuracy of responses, used the same dissemination methods, and recruited relatively large samples of participants with the same demographic characteristics. Nevertheless, the study results do not include longitudinal data, and this limitation should be considered when making comparative statements. The structural equation modeling approach in the present work allowed simultaneous examination of relationships among multiple predictors and multiple latent outcomes while controlling for measurement error. Many significant regression paths were uncovered; however, in some cases, the strength of the relationships was weak.

In times of uncertainty and fear, such as this pandemic, there is a need to identify population weaknesses and strengths, as well as psychological and mental influences. Such findings will help stakeholders develop interventions and strategies to maintain or improve healthy and effective mental health. Based οn the findings of this research, the fοllοwing recommendations are provided to policymakers to maximize the public health impact of managing the psychοlοgical impact of the pandemic: 1) examine and monitor the psychological impact in the general pοpulatiοn and in specific subgrοups, as these may change with the ongoing pandemic, 2) develop and implement psychosocial programs to facilitate social reintegration and regulate emotional difficulties for high-risk groups, 3) promote psychosocial programs for the general population to improve emotional resilience and coping skills, 4) implement targeted campaigns for groups with low adherence to precautions against COVID -19.

Even though we are at a stage where we have the virus well under control, we are still deregulating its effects. Five months after the isolation measures ended, it was clear that the pandemic continued to affect certain vulnerable groups. Today’s research, focused on the COVID -19 syndrome, should take into account that patients’ emotional difficulties may overlap with the effects of the stringent first-wave measures. The COVID -19 pandemic was a major stressor for people worldwide, and the psychological impact of the pandemic may continue to affect people’s health and well-being after the acute phase. Psychological factors such as anxiety, depression, and stress can have a significant impact on a person’s overall health and well-being and contribute to the development and persistence of long-lasting COVID symptoms. Therefore, it is important to consider the psychological impact of the pandemic during the first wave when examining long-term COVID symptoms.
